# The Antiviral Effects of Na,K-ATPase Inhibition: A Minireview

**DOI:** 10.3390/ijms19082154

**Published:** 2018-07-24

**Authors:** Luciano Amarelle, Emilia Lecuona

**Affiliations:** 1 Division of Pulmonary and Critical Care, Feinberg School of Medicine, Northwestern University, Chicago, IL 60611, USA; luciano@northwestern.edu; 2 Departamento de Fisiopatología, Hospital de Clínicas, Facultad de Medicina, Universidad de la República, Montevideo 11600, Uruguay

**Keywords:** cardiac glycosides, virus, Na,K-ATPase, antiviral treatment

## Abstract

Since being first described more than 60 years ago, Na,K-ATPase has been extensively studied, while novel concepts about its structure, physiology, and biological roles continue to be elucidated. Cardiac glycosides not only inhibit the pump function of Na,K-ATPase but also activate intracellular signal transduction pathways, which are important in many biological processes. Recently, antiviral effects have been described as a novel feature of Na,K-ATPase inhibition with the use of cardiac glycosides. Cardiac glycosides have been reported to be effective against both DNA viruses such as cytomegalovirus and herpes simplex and RNA viruses such as influenza, chikungunya, coronavirus, and respiratory syncytial virus, among others. Consequently, cardiac glycosides have emerged as potential broad-spectrum antiviral drugs, with the great advantage of targeting cell host proteins, which help to minimize resistance to antiviral treatments, making them a very promising strategy against human viral infections. Here, we review the effect of cardiac glycosides on viral biology and the mechanisms by which these drugs impair the replication of this array of different viruses.

## 1. Introduction

Since first described by Skou more than 60 years ago [1], Na,K-ATPase has been extensively studied, and novel concepts about structure, physiology, and biological roles continue to appear. The classical role of Na,K-ATPase is to maintain the electrolyte homeostasis of the cells by pumping cations in and out of the cell using the energy obtained from the hydrolysis of (Adenosine triphosphate) ATP. However, Na,K-ATPase is also a key scaffolding protein that is able to interact with signaling proteins such as protein kinase C (PKC) and phosphoinositide 3-kinase (PI3K) [2,3,4,5]. It also works as a classical receptor in which the binding of cardiac glycosides induces activation of the tyrosine kinase Src and down-stream signaling cascades, independent of changes in intracellular ions [6,7,8]. Viruses are intracellular parasites whose life cycle is dependent on hijacking cellular functions like protein synthesis and intracellular transport of molecules to promote their replication and spreading. The infection begins with the attachment of the viral particle to surface-exposed cellular molecules and their entry to the cell by endocytosis. Once the viral components are in the intracellular environment and the genome is transcribed, the viral proteins are synthesized using the host cell translational machinery, and the new viral particles are transported to the surface to be released and infect other cells [9]. Viruses are very frequent causative agents of human infectious diseases and cancer, and their treatment is often challenging, as resistance to antiviral drugs has been reported for many important pathogens such as influenza, hepatitis B, and herpes simplex virus [10,11,12,13]. Targeting host cell components such as Na,K-ATPase is a very promising antiviral strategy in order to minimize the resistance to antiviral drugs, and has been shown to be effective in a broad spectrum of viral species. In this review, we analyze both the effects that viral infection have on the Na,K-ATPase function and the effect of Na,K-ATPase ligands cardiac glycosides on viral biology. We will also review the mechanisms by which these drugs impair the replication of different types of viruses (Table 1).

## 2. Na,K-ATPase Function Altered by Viral Infection

Na,K-ATPase function can be directly affected by both DNA and RNA viruses. Human parvovirus B19 is a DNA virus with tropism for erythroid progenitor cells causing erythema infectiosum (fifth disease) in children and other pathologic conditions such as arthropathies and pure red blood cell aplasia [29]. The expression of human parvovirus B19 capsid protein VP1 decreases Na,K-ATPase activity in *Xenopus* oocytes partially due to the VP1 phospholipase A2 activity dependent formation of lysophosphatidylcholine [30]. In addition, Chiu et al. found that recombinant VP1 can also decrease Na,K-ATPase expression in A549 cells [31]. Infection by RNA viruses can also affect the expression and activity of the Na,K-ATPase. Na,K-ATPase is downregulated in alveolar epithelial cells infected with influenza A H1N1 and H5N1strains, affecting alveolar fluid clearance [32]. In addition, influenza A virus infection induces decreased expression of Na,K-ATPase in the plasma membrane of alveolar epithelial cells with paracrine factors released from infected cells [33]. Na,K-ATPase activity can also be decreased by sindbis virus [34] and enterovirus coxsackie B infection [35], causing important changes in the intracellular concentration of potassium and sodium and consequently in membrane potential [36]. Interestingly, enterovirus 71 (EV71), agent of hand- foot-and-mouth disease (HFMD) in pediatric population, interacts with the β3 subunit of the Na,K-ATPase causing an increase of its expression [37].

## 3. DNA Viruses Affected by Na,K-ATPase Modulation

Cardiac glycosides are a family of steroidal compounds commonly used in the treatment of cardiac diseases. These glycosides inhibit the Na,K-ATPase pump function, resulting in changes in the intracellular concentrations of sodium, potassium, and calcium [38], and also trigger signaling transduction pathways at low concentrations [14]. It is not well understood whether the action of cardiac glycosides on viral replication is due to changes in ion homeostasis, or by activation of intracellular signaling pathways; however, these compounds are effective on a diversity of viruses, from which we infer that there are mechanisms affecting host processes that are crucial for viral replication. Cardiac glycosides inhibit cytomegalovirus (CMV) replication, a herpesvirus causative of important human diseases, at nanomolar concentrations [15], with an additive effect when combined with antiviral drugs for CMV as ganciclovir [16]. In a recent publication by Cohen et al., a panel of cardiac glycosides was used to study its efficacy against CMV in human lung fibroblasts, and it was found that the inhibitory activity on CMV replication was due to a decrease in viral protein translation, and that the antiviral potency depended on the structure of the cardiac glycosides and its specific interaction with the Na,K-ATPase [17]. Cardiac glycosides can also be effective on other DNA viruses such as herpes simplex virus (HSV) by inhibiting the expression of viral genes, with the antiviral action correlated with the potency against the Na,K-ATPase [18]. In addition, Su et al. have reported that digitoxin inhibits HSV replication with a 50% effective concentration (EC_50_) of 0.05 µM, while the EC_50_ for classical anti HSV drugs (acyclovir and ganciclovir) is higher than 1.5 µM. Digitoxin impaired the HSV viral life cycle at two different steps: viral DNA synthesis and viral release form the host cells. The authors also showed that others cardiac glycosides such as digoxin, ouabain, and G-strophanthin have comparable anti-HSV activity [19]. Finally, adenoviruses, which are common human pathogens, are also susceptible to cardiac glycosides such as digitoxin and digoxin, which are able to impair adenovirus genome replication by altering the host pre-RNA splicing machinery [39].

## 4. RNA Viruses Affected by Na,K-ATPase Modulation

A diversity of RNA viruses is vulnerable to cardiac glycosides treatment. Chikungunya virus, the agent of a human epidemic mosquito-borne disease [20], is susceptible to treatment with cardiac glycosides. Ashbrook et al. screened a library of small molecules for the capacity to modulate chikungunya virus infection in human osteosarcoma cells and found that digoxin has antiviral activity on chikungunya and other alphaviruses (including river virus, sindbis virus, and vesicular stomatitis virus) by impairing the viral cycle at post entry steps via inhibition of Na,K-ATPase [40]. Moreover, other RNA viruses are affected by Na,K-ATPase inhibition. Coronaviruses, which cause intestinal and respiratory diseases and are responsible for middle-east respiratory syndrome (MERS-CoV) and epidemic severe acute respiratory syndrome (SARS-CoV) in humans [21], are repressed when the Na,K-ATPase α1-subunit is silenced or inhibited by low dose of cardiac glycosides, an effect caused by an impairment of the virus entry at an early stage. Importantly, the cardiac glycosides’ antiviral effect is relieved by inhibition of the Src pathway [22]. Transmissible gastroenteritis coronavirus (TGEV) is also susceptible to ouabain and digitoxin [41]. The respiratory syncytial virus (RSV) is a common cause of seasonal respiratory disease in children, which can lead to severe respiratory failure, contributing to hospitalization and mortality in the pediatric population [23]; no specific antiviral treatment or vaccine is yet available. Ouabain at a concentration of 10 nM inhibits RSV replication in an in vitro model [42]. Also, ebola virus (EV), which triggers severe hemorrhagic fever in humans with a high mortality rate, can be inhibited by cardiac glycosides. Na,K-ATPase was identified in a proteomic analysis of EV interaction with host cell proteins, and nanomolar concentrations of ouabain were able to impair the viral replication by decreasing the levels of viral RNA [24]. Additionally, this finding was confirmed by a screening of compounds with activity against EV [25]. Influenza is an infectious disease caused by various types of influenza virus, and it is characterized by a highly contagious, acute respiratory syndrome that carries significant morbidity and mortality worldwide. Many influenza strains have developed pharmacological resistance to available antiviral drugs, and the development of new anti-influenza treatments has been of great interest in recent years. Pharmacological screening studies have highlighted the potential efficacy of cardiac glycosides as anti-influenza drugs. A high-throughput screen made by Hoffmann et al. found that nanomolar doses of Na,K-ATPase inhibitors, such as ouabain and lanatoside C, exert inhibition on influenza virus replication in vitro [43]. Moreover, the β1 subunit of the Na,K-ATPase interacts with the M2 and BM2 influenza A proteins and their knockdown decreases influenza virus replication [26]. We have also observed that the inhibition of Na,K-ATPase by cardiac glycosides decreases influenza virus replication by inhibiting the host cell translational machinery via a decrease in intracellular potassium [44]. Consistently with this, other authors have shown that ouabain does not affect the budding rate of the influenza virus in MDCK (Madin-Darby Canine Kidney) infected cells [45]. Interestingly, human immunodeficiency virus (HIV) can also be susceptible to cardiac glycosides treatment. HIV infection is a worldwide spread infectious disease affecting more than 30 million people around the globe that can lead to acquired immunodeficiency syndrome or AIDS if not treated, increasing the risk for opportunistic infections and some types of cancer [46]. In a screening study by Laird et al., different members of the cardiac glycosides family were shown to be active against HIV-1 [27]; moreover, Wong et al. demonstrated that digoxin inhibits the replication of clinical strains of HIV-1 by impairing the splicing mechanism of viral RNA and consequently diminishing the structural protein synthesis of the virus [28]. Recent work published by the same group reported that low concentrations of cardiac glycosides inhibit HIV-1 gene expression through modulation of the mitogen-activated protein kinases/extracellular signal-regulated kinases (MEK1/2-ERK1/2) signaling via interaction with the Na,K-ATPase, independent of alterations in intracellular calcium [47].

## 5. Mechanisms of Cardiac Glycosides Antiviral Effect

Although there is strong in vitro evidence of the antiviral activity of Na,K-ATPase modulation, little is known about the intracellular mechanisms leading to this effect. What seems to be clear is that either by activating signaling cascades or by altering the concentration of intracellular ions, the binding of cardiac glycosides to Na,K-ATPase determines an environment that is unfavorable for viral replications. The main antiviral mechanisms suggested for both DNA and RNA viruses are decreased transcription of viral genes and impaired synthesis of viral proteins by interfering with the host translational machinery. For instance, the replication of HSV is not affected by pre-treatment with high doses of digitoxin but via a reduction of the genomic DNA transcription at 10 h post infection [19]. Interestingly, digitoxin also inhibits the viral release of HSV, thereby acting on two different stages of the viral cycle [19]. Concordantly, Kapoor et al. showed that another herpesvirus, the human CMV, is also susceptible to cardiac glycosides via an inhibition of viral DNA replication. They described reduced levels of nuclear factor κB (NF-κB) 12 h post-infection and decreased expression of intermediate-early viral proteins IE1 and IE2. The authors hypothesize that the inhibitory mechanism might be mediated via an interaction between Na,K,ATPase and other ion channels like the voltage-gated K^+^ channel hERG, whose expression is induced by CMV infection and downregulated by cardiac glycoside treatment [15]. CMV protein synthesis has also been shown to decrease in cells treated with the cardiac glycoside convallatoxin. The mechanism proposed is a decreased transport of the essential amino acid methionine without affecting the viral mRNA accumulation; however, the authors did not study further mechanisms or links between Na,K-ATPase modulation and the altered amino acid transport [17]. Wong et al. studied the mechanism by which cardiac glycosides are active against HIV. The authors observed that digoxin inhibits HIV replication by impairing the alternative splicing of viral pre-mRNA needed for the synthesis of HIV proteins, therefore reducing the viral gene expression. This mechanism was confirmed using HIV-1 clinical strains in human CD4+ cells. In addition, the authors observed that digoxin selectively inhibits the key regulatory protein Rev [48], leading to impaired export of Rev-dependent mRNAs that produce viral structural proteins. Although the mechanism by which cardiac glycosides interferes with the host alternative splicing machinery remains unclear, they proposed that digoxin-induced alteration of signaling cascades affects the activity of factors known to regulate HIV-1 RNA splicing. These factors include the kinase proteins SR, which have been shown to be modulated by digoxin [28]. Moreover, in a recent publication these authors reported that cardiac glycosides from both families, cardenolides (vertebrate-derived) and bufadenolides (plant-derived), have anti-HIV activity in vitro and ex vivo in HIV-infected T cells from humans with inhibitory effects at the nano-molar range. Importantly, the inhibition of HIV-1 gene expression by these drugs is independent of changes in Ca^2+^, PI3K-AKT, and JNK/p38 mitogen-activated protein kinases (MAPK). Src activation by cardiac glycosides can trigger the classical MAPK (extracellular signal-regulated kinase (ERK) 1/2) signaling through the Raf-MEK cascade, which has been observed to inhibit HIV-1 gene expression, highlighting the importance of intracellular signaling triggered by cardiac glycoside binding as a potential therapeutic strategy for HIV [47]. Similarly, Grosso et al. observed that adenovirus, which also depends on the host RNA splicing machinery, is susceptible to cardiac glycoside treatment. The authors found that digoxin and digitoxin affect the synthesis of viral proteins by reducing the expression of late viral protein mRNAs through altering the E1A RNA splicing (favoring the production of E1 RNA isoform 13S over 12S) early in infection and partially blocking the transition from E1 RNA isoform 12S and 13S to 9S at late stages of virus replication [39]. Src signaling is also reported to be involved in the inhibition of coronavirus and vesicular stomatitis virus infections, as the inhibition of the virus can be rescued by inhibitors of Src. Cardiac glycosides were proposed to affect the entry stage of the virus by interfering with endocytosis by a non-elucidated pathway. Interestingly, influenza virus infection was not affected by this mechanism [22]; indeed, our results show that influenza virus is impaired by ouabain at nano-molar concentrations by inhibiting the translation of viral proteins through a decrease in the concentration of intracellular potassium [44]. 

## 6. Conclusions

Na,K-ATPase is a ubiquitous transmembrane protein that not only pumps ions in and out of the cell but also plays an important role in intracellular signaling processes. Due to its effect on Na,K-ATPase, the use of cardiac glycosides as antiviral drugs is very promising. The strong inhibitory effects of these drugs occur at different levels of the life cycle of different virus species. However, in the publications that profoundly studied the mechanism of action of cardiac glycosides as antiviral drugs, the main findings correlate with either an impairment on the viral genome replication or a decrease in the viral mRNA or protein synthesis, suggesting that this drugs target host processes that are essential for the viruses to accomplish a successful replication cycle. These mechanisms should be further explored in order to develop novel antiviral treatments with two very important advantages: low risk of resistance and broad spectrum of action.

## Figures and Tables

**Table 1:** Summary of cardiac glicosides with reported antiviral activity.

Viruses	Components with Antiviral Activity	Mechanism of Action Proposed	References
**DNA Viruses**			
Cytomegalovirus	digoxin, ouabain, digitoxin, convallatoxin	Inhibition of viral protein translation	[14,15,16]
Herpes simplex virus	ouabain, digoxin, digitoxin, G-strophanthin	Inhibition of viral gene expression	[17]
		Inhibition of viral release	[18]
Adenovirus	digoxin, digitoxin	Alteration of viral pre-RNA splicing	[19]
**RNA Viruses**			
Chikungunya virus	digoxin		[20]
Coronaviruses	ouabain, digitoxin	Inhibition of Src pathway	
		Inhibition of viral entry	[21,22]
Respiratory syncytial virus	ouabain		[23]
Ebola virus	ouabain	Alteration of viral RNA	[24]
Influenza virus	ouabain, digoxin, lanatoside C	Inhibition of viral protein translation	[25,26]
Human immunodeficiency virus	digoxin, ouabain, lanatoside C, digitoxin	Alteration of viral pre-RNA splicing	[27,28]
